# Clinical evaluation of ocular biometry of dual Scheimpflug analyzer, GALILEI G6 and swept source optical coherence tomography, ANTERION

**DOI:** 10.1038/s41598-022-07696-1

**Published:** 2022-03-04

**Authors:** Boonsong Wanichwecharungruang, Anyarak Amornpetchsathaporn, Kittipong Kongsomboon, Wisakorn Wongwijitsook, Kornkamol Annopawong, Somporn Chantra

**Affiliations:** 1 Department of Ophthalmology, Rajavithi Hospital and Rangsit Medical College, 2, Phayatai Road, Bangkok, 10400 Thailand; 2 Department of Ophthalmology, Priest Hospital, Bangkok, Thailand; 3 Department of Preventive and Social Medicine, Faculty of Medicine, Srinakharinwirot University, Nakhon Nayok, Thailand

**Keywords:** Health care, Medical research

## Abstract

To evaluate the performance of a new swept source optical coherence tomography optical biometer, ANTERION, in ocular biometry and intraocular lens (IOL) calculation compared with the reference standard of Dual Scheimpflug Analyzer (GALILEI, G6). A prospective comparative study was conducted in a tertiary eye center. Cataract patients were scanned with both devices in a random fashion, and parameters from the devices were analyzed in terms of mean difference and intraclass correlation coefficient (ICC). Bland–Altman plots were performed to compare agreement between the devices. Ninety-six eyes from 96 patients were enrolled for evaluation. With the exception of ACD, all parameters were significantly different, but excellent agreement was revealed for all of them. The mean difference in axial length was 0.03 mm, and ICC was 0.999. Calculated IOL power with Barrett formula revealed that 93.75% were within 1 diopter and the prediction error was 0.03 diopter. Biometry of the devices were arithmetically different. However, the mean difference of the key factors in IOL calculation were small and appeared to be negligible for the purposes of clinical application. The performance of ANTERION was comparable to that of G6 in biometric measurement and IOL calculation; however, the devices cannot be used interchangeably.

## Introduction

One of the key aims in cataract surgery is to ensure that patients get the correct intraocular lens (IOL) implantation. Various types of IOL, including monofocal, multifocal, toric, and enhanced depth-of-focus, have been developed to provide the expected vision. Accuracy of IOL calculation, obtained with a proper ocular biometer, is essential for the success of the procedure.

Originally, the ultrasound (US) biometer was a gold standard for IOL calculation. The US system requires ocular contact to obtain ocular structure for biometric measurement of anterior chamber depth (ACD), lens thickness (LT) and axial length (AL). The IOL calculation formula of the US system is based on the parameters of AL and keratometry (K). The contact manner of the US system requires a skillful operator and a very co-operative patient during IOL measurement. US is useful for dense cataract biometry; however, it is rather inconvenient for clinical practice.

Optical biometers have been evolving in recent decades. The optical systems can be performed in a non-contact manner, and they are more convenient than the US one. They have been widely used and have become the gold standard for IOL calculation. Anterior segment parameters and axial length (AL) are crucial factors in IOL power calculation. A few systems are available to determine those parameters, such as Placido disc, Scheimpflug camera and OCT, and these systems can be combined in one device, for instance, the GALILEI Dual Scheimpflug Analyzer (GSA)^1^, which demonstrates good repeatability in anterior segment evaluation with high accuracy of post-operative refraction after phacoemulsification and IOL implantation (PEI)^2^. In recent years, we have used GSA version GALILEI G6 (Ziemer Ophthalmic Systems AG Port, Switzerland) as the main IOL biometer in our clinic.

Swept source OCT (SS-OCT) is a new system of biometric measurement. Its performance appears to be superior to that of the previous platform of time domain OCT (TD-OCT) in penetration of dense cataracts^3,4^, and it has recently been gradually gaining in popularity. ANTERION, a newer SS-OCT, is currently employed in many clinical practices^2^, and evaluation of this new device is necessary before we use it in real-world practice. Therefore, we investigated the performance of ANTERION, in comparison with that of G6, in terms of biometric measurement and IOL calculation for cataract patients.

## Methods

We conducted a prospective comparison of ANTERION and G6 in biometric measurement and IOL calculation for cataract patients in a tertiary eye care center at Rajavithi Hospital. The research protocol was approved by the Ethics Committee of Rajavithi Hospital, Ministry of Public Health, in December 2020, and the study was performed between January and May 2021, in accordance with all of the guidelines of the Ethics Committee for experimental investigation in human subjects. All investigations were carried out in accordance with the provisions of the Declaration of Helsinki, and informed consent was read and signed by all participants. Informed consent for publication of identifying information/images in an online open-access publication was obtained from participant.

### Inclusion and exclusion criteria

We recruited patients from the general ophthalmic clinic of the hospital. Inclusion criteria were patients aged ≥ 40 years old who had cataract with or without visual symptoms. Patients who were able to undergo a complete examination were enrolled in the study.

Exclusion criteria included patients who had opaque optical media, dense cataract, lens subluxation, anterior and/or posterior segment diseases such as advanced pterygium, uveitis, diabetic retinopathy, or maculopathy. Participants who had previous history of ocular trauma, ocular surgery (such as refractive surgery), glaucoma (trabeculectomy and/or glaucoma tube shunt), or vitreoretinal surgery were also excluded.

### Ocular examinations

Ocular examination including Snellen visual acuity with logMar conversion, autorefraction (RC-5000, Tomey, Japan), slit lamp, pneumatic tonometry, and dilated fundus ophthalmoscopy were performed by one of the experienced ophthalmologists (BW, WW, and SC).

### The devices: dual Sheimpflug analyzer and SS-OCT

G6 employs a Placido disc and dual Scheimpflug camera to determine keratometry, central corneal thickness (CCT) and anterior chamber depth (ACD). G6’s two rotating cameras are capable of scanning both sides of the cornea with minimized decentration due to saccadic eye movement. In addition, built-in Placido disk topography assesses the corneal topography more accurately^5^. G6 combines a 880 nm light source with optical low coherence interferometry (OLCI) to determine the lens thickness (LT) and AL.

SS-OCT applies a tuneable light source, namely swept source, to a beam splitter, sending one arm of the scanning beam to the ocular structure and the other to the reference mirror. Light from both beams interferes at the beam splitter and is detected by a fast point detector. The interferometric fringes are Fourier-transformed to generate the depth profile (A-scan) and cross-sectional image (B-scan), which allows determination of ocular dimensions^6^. SS-OCT does not require a moving reference mirror as in TD-OCT; hence, it is capable of obtaining a higher A scan rate and has a shorter acquisition time. Moreover, advances in wavelength-tuneable lasers allow for a finer resolution than that of TD-OCT. ANTERION utilises a 1300 nm light source with 50,000 A scan/sec, capturing 32 mm scan depth, 16.5 mm scan width, axial resolution < 10 microns and lateral resolution of 30–45 microns^6^.

### Scanning protocols

ANTERION (Software on HEYEX 2.5.5, Heidelberg Engineering GmbH, Germany) and GALILEI (V6.4.2 Ziemer, Port, Switzerland) were scanned 10 min apart in random sequences by trained technicians in a standard illuminated room. No pupillary dilation was required. Image quality was checked by a research fellow (AP) who had 1 year experience of using both devices. Biometric parameters of ACD, AL, LT, K1, K2, astigmatism, and white-to-white corneal diameter (WTW) were recorded in Excel and transferred to SPSS (Version 22, SPSS Inc., IBM, Chicago, USA) for analysis.

### Intraocular lens calculation

We applied the required biometric parameters, ACD, K1, K2, AL, WTW, and LT of both devices to Barrett Universal II formula for IOL calculation (https://calc.apacrs.org/barrett_universal2105/, accessed September 25, 2021). We used A constant 119.0 of the IOL model SN60WF (Alcon Laboratories, Fort Worth, TX). The reports included IOL power (diopter, D) and prediction error (D) of those eyes.

### Outcome measurement and statistical analyses

Kolmogorov–Smirnov test was analyzed for normal data distribution. Comparisons of the biometric parameters were made with independent *t*-test for parametric datasets and Mann–Whitney *U* test for non-parametric datasets. Statistical significance was set at *p* < 0.05.

The main outcome measure was the agreement of the biometric parameters of the 2 devices. Intraclass correlation coefficient (ICC) and confidence limit were analyzed and classified as follows: < 0.4 was poor; 0.4–0.59 was fair; 0.60–0.74 was good; and 0.75–1.00 indicated excellent agreement^7^. Bland–Altman was plotted to demonstrate the concordance between the devices in relation to IOL power and prediction error.

## Results

One hundred and two patients who visited the general ophthalmic clinic were initially enrolled in the study. G6 failed to measure AL in two patients, one of whom had diagnosis of primary angle-closure glaucoma, and the other of whom had senile cataract (SC) with punctate epithelial erosion. ANTERION failed to measure AL in four patients with diagnosis of SC (1); primary-angle closure glaucoma (1); SC with Meibomian gland dysfunction; (1) and SC with pterygium (1). Ninety-six eyes from 96 patients were eligible for statistical analysis. Mean age, visual acuity, spherical equivalent and intraocular pressure (IOP) are displayed in Table 1.

**Table 1:** Number of patients and demographic data.

Data	Number
**Number of patients**	96
Male	35 (36.5%)
Female	61 (63.5%)
**Number of eyes**	96
Right	50 (52.1%)
Left	46 (47.9%)
Age (years old)	62.69 ± 8.84
VA (logMAR)	0.31 ± 0.39
Spherical equivalent (D)	0.03 ± 2.08
Intraocular pressure (mmHg)	16.74 ± 3.28

All measured parameters and calculated IOL powers, with the exception of ACD, revealed significant differences between the devices. The AL Mean difference was − 0.03 ± 0.03 mm; however, the intraclass correlation coefficient (ICC) between the two devices of all parameters showed excellent agreement (Table 2).

**Table 2:** Comparison and Intraclass correlation coefficient of various biometric parameters between GALILEI G6 and ANTERION.

Parameter	GALILEI G6	ANTERION	Mean difference ± SD	*p* value	ICC	Confident limit	
	Mean ± SD	Mean ± SD				lower	upper
ACD (mm)	2.93 ± 0.36	2.94 ± 0.38	0.00 ± 0.09	0.630^a^	0.970	0.954	0.980
AL (mm)	23.30 ± 0.99	23.32 ± 0.99	− 0.03 ± 0.03	< 0.001^a^	0.999	0.996	1.000
K1 (D)	43.61 ± 1.56	43.34 ± 1.53	0.27 ± 0.46	< 0.001^a^	0.942	0.859	0.970
K2 (D)	44.65 ± 1.58	44.33 ± 1.56	0.32 ± 0.45	< 0.001^a^	0.939	0.807	0.972
LT (mm)	4.70 ± 0.36	4.85 ± 0.37	− 0.15 ± 0.15	< 0.001^a^	0.849	0.335	0.943
WTW (mm)	11.81 ± 0.40	11.66 ± 0.47	0.15 ± 0.21	< 0.001^a^	0.835	0.570	0.921
IOL power (D)	21.33 ± 2.63	21.55 ± 2.67	− 0.22 ± 0.61	0.001^b^	0.971	0.951	0.982

Values are presented as mean ± SD.

^a^*p*-value from independent *t*-test .

^b^*p*-value from Mann–Whitney *U* test .

**Figure 1:**
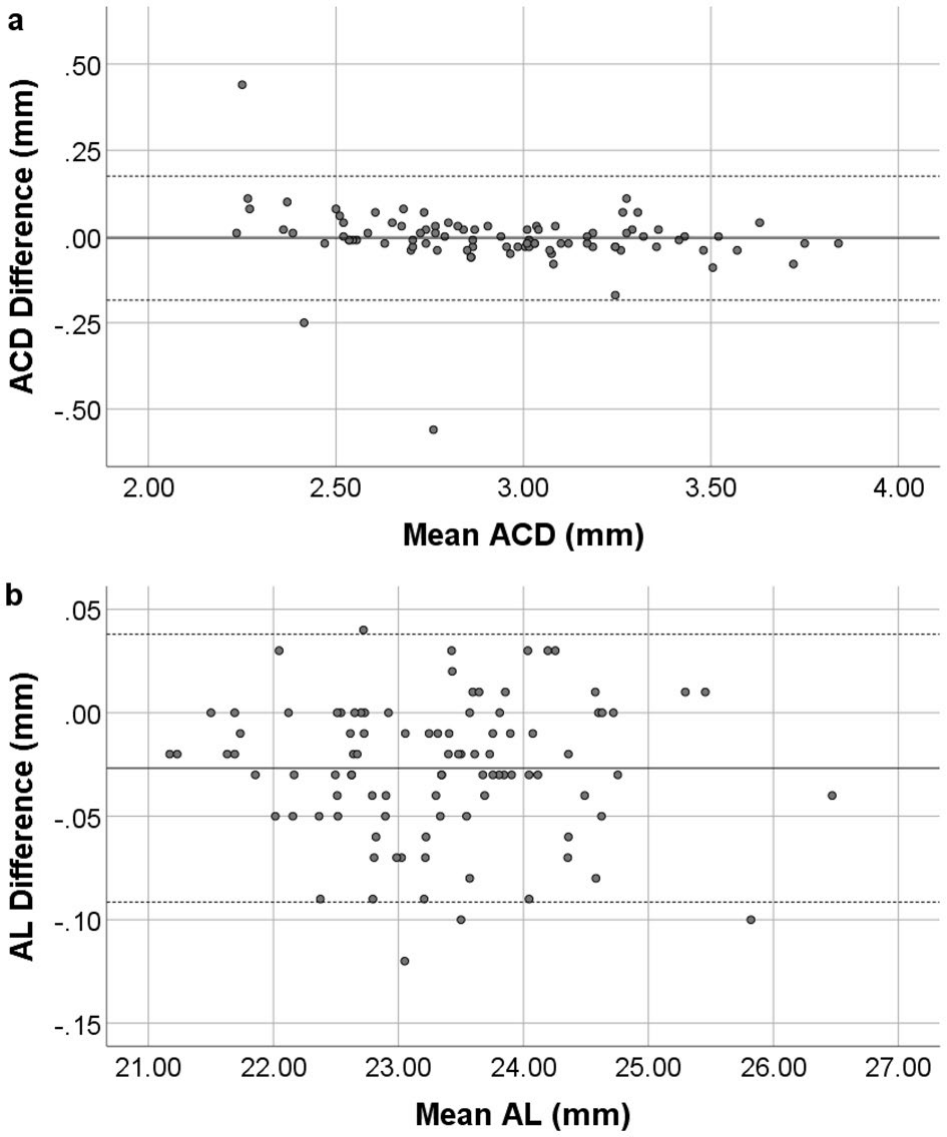
Bland–Altman plot of the ACD (**a**) and AL (**b**) from GALILEI G6 and ANTERION. The solid line represents the mean difference, whereas the dotted lines on each side show the upper and lower 95% LoA. The ACD and AL plot demonstrate that only three and four eyes respectively of the total of 96 eyes were out of the 95% LoAs, indicating excellent agreement in ACD and AL between the two devices. Created by SPSS (Version 22, SPSS Inc., IBM, Chicago, USA).

The ACD and AL differences between the devices are shown with Bland–Altman plot, Fig. 1.

**Figure 2:**
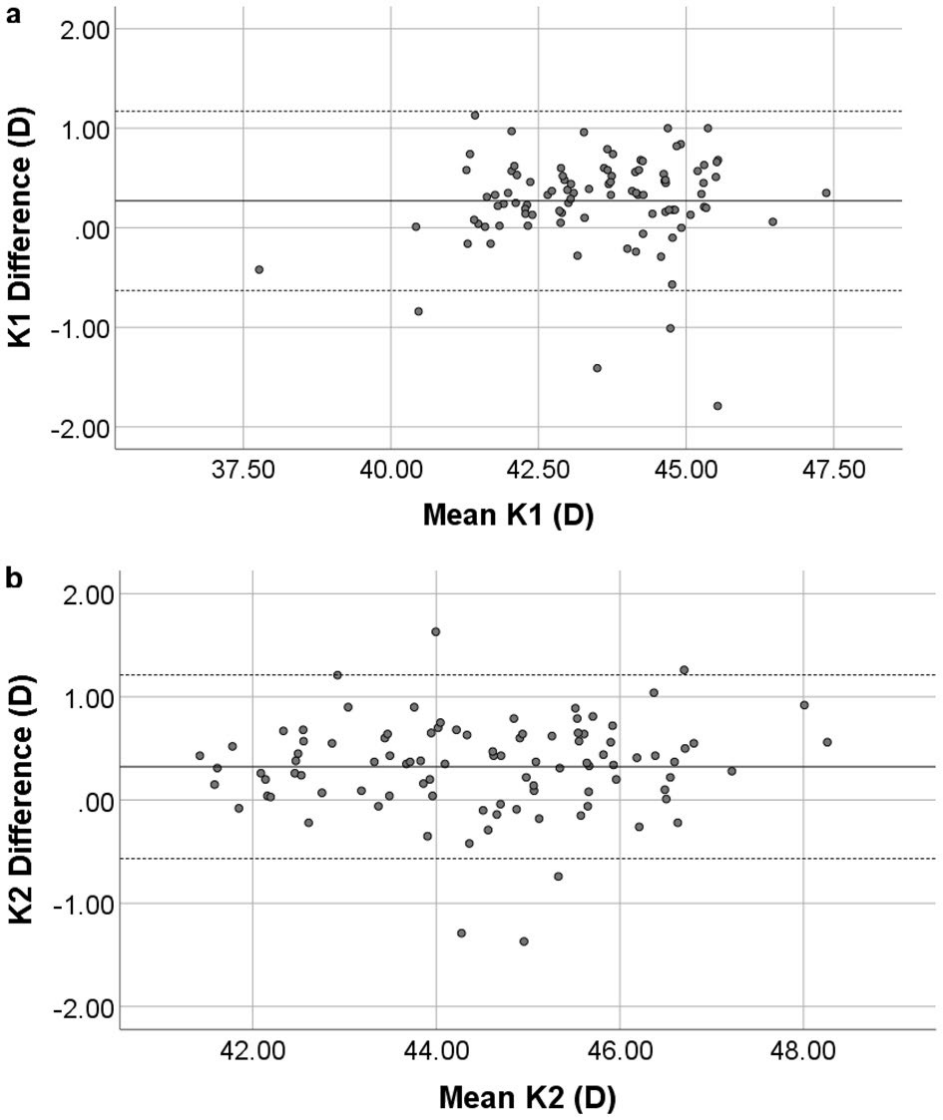
Bland–Altman plot of K1 (**a**) and K2 (**b**) from GALILEI G6 and ANTERION. The solid line represents the mean difference, while the dotted lines on each side show the upper and lower 95% LoA. The K1 and K2 plot demonstrate that only four and five eyes respectively of the total 96 eyes were out of the 95% LoAs, indicating excellent K1 and K2 measurement agreement between the two devices. Created by SPSS (Version 22, SPSS Inc., IBM, Chicago, USA).

The keratometry, K1 and K2, differences between the devices are shown with Bland–Altman plot, Fig. 2.

**Figure 3:**
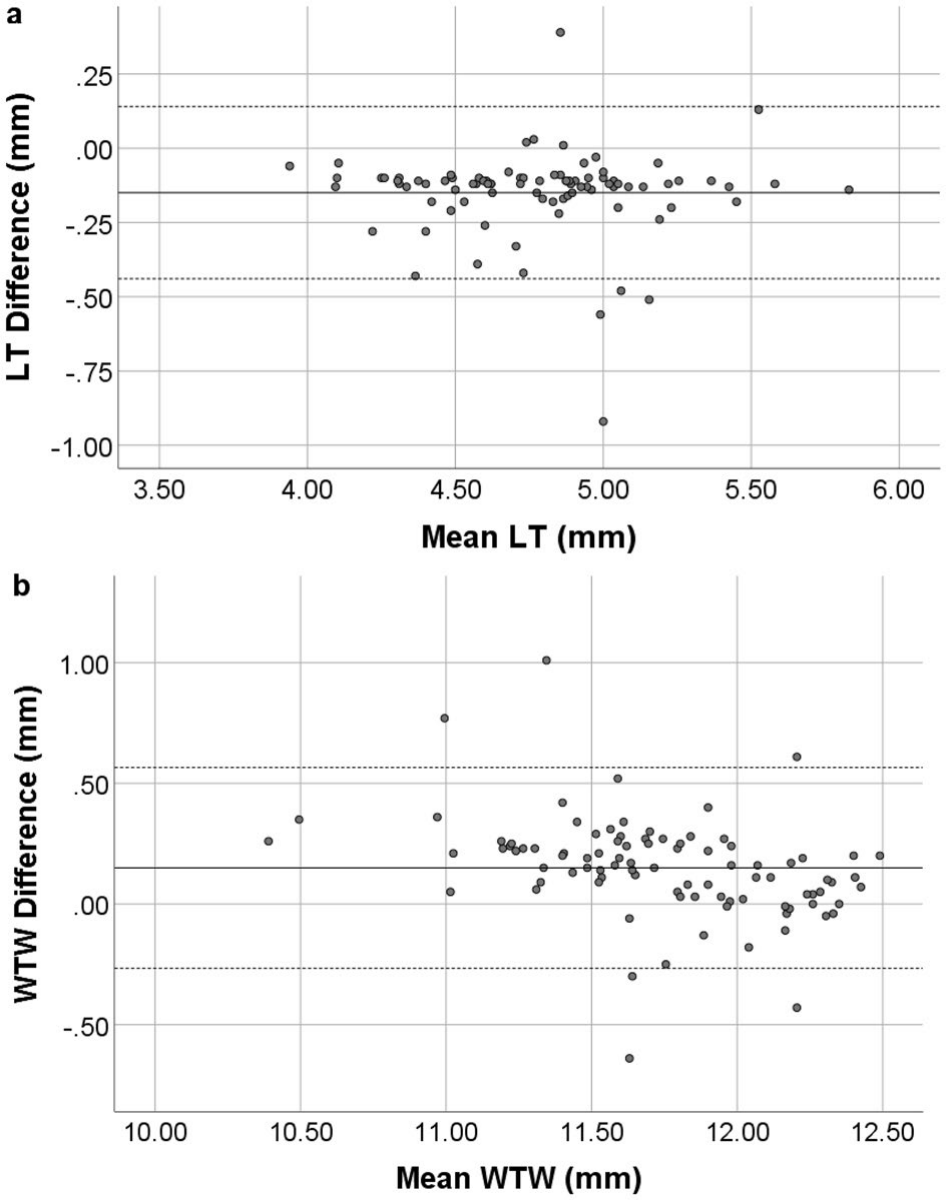
Bland–Altman plot of the LT (**a**) and WTW (**b**) from GALILEI G6 and Anterion. The solid line represents the mean difference, whereas the dotted lines on each side show the upper and lower 95% LoA. Both LT and WTW plot demonstrate that only six eyes from the total of 96 were out of the 95% LoAs, indicating excellent concordance in LT and WTW calculation between the two devices. Created by SPSS (Version 22, SPSS Inc., IBM, Chicago, USA).

Lens thickness and WTW differences between the devices are shown with Bland–Altman plot, Fig. 3.

### Intraocular lens calculation

**Figure 4:**
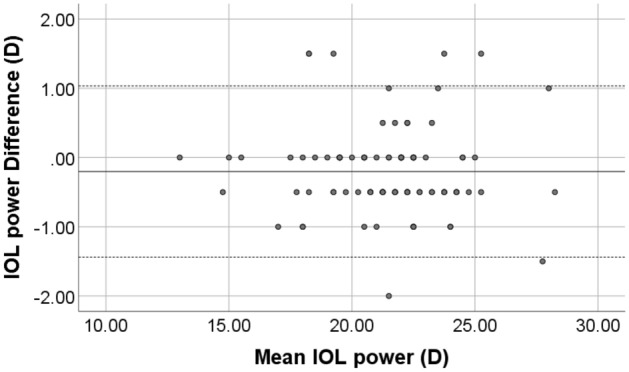
Bland–Altman plot of the IOL power using parameters from GALILEI G6 and ANTERION. The solid line represents the mean difference, whereas the dots on each side show the upper and lower 95% LoA. The plot demonstrates that six eyes at IOL power difference of ± 1.5 D (5 eyes) and 2 D (1 eye) from the total of 96 eyes were out of the 95% LoAs, indicating excellent agreement between the two devices’ measurement of IOL power. Created by SPSS (Version 22, SPSS Inc., IBM, Chicago, USA).

The IOL power differences between the devices are shown with Bland–Altman plot, Fig. 4.

The IOL power obtained from the two devices revealed that 35 eyes (36.5%) had the same IOL power, 41 eyes (42.7%) had ± 0.5 D difference, 14 eyes (14.6%) had ± 1.0 D difference, five eyes (5.2%) had ± 1.5 D difference, and only one eye (1%) had ± 2.0 D difference.

### Prediction error

**Figure 5:**
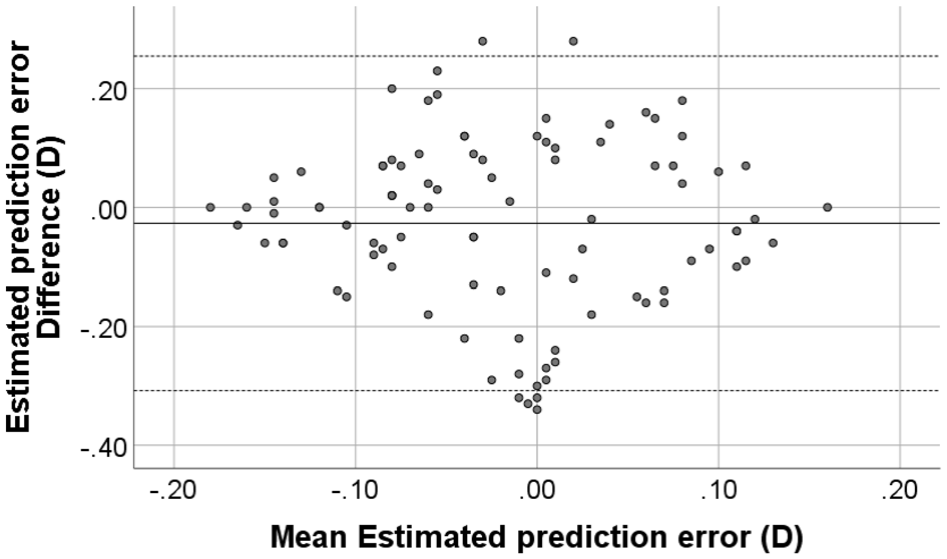
Bland–Altman plot of the prediction error using parameters from GALILEI G6 and ANTERION. The solid line represents the mean difference, while the dotted lines on each side show the upper and lower 95% LoA. The plot reveals that six eyes were out of the 95% LoAs. Created by SPSS (Version 22, SPSS Inc., IBM, Chicago, USA).

Prediction error represents the estimated post-operative residual refraction, shown in the report of IOL calculation formula, next to the calculated IOL powers. There was no statistical difference between the prediction errors calculated by the two devices. Mean prediction error from ANTERION was 0.01 ± 0.11 D while that of G6 was 0.03 ± 0.10 D, with mean difference of 0.03 ± 0.14 D (*p* = 0.165). A Bland–Altman plot comparing the prediction error obtained from each device is displayed in Fig. 5.

## Discussion

Biometry is an invaluable tool for use with cataract patients who are candidates for PEI. Both GALILEI G6 and ANTERION have been proven to perform well in ocular biometry with anterior segment and AL. Ladi et al. reported the coefficient of variation (COV) for repeatability and reproducibility of CCT obtained by GSA of 0.43% and 0.377% respectively^8^. Wang et al. reported COV of 0.1–0.35%, ICC > 0.996 in corneal power measurement^9^ and good accuracy of mean absolute prediction error (MAE) of IOL power, within 1 D in 93% of cases^10^. Savini et al. reported high repeatability of corneal power, anterior chamber angle measurement^11,12^ and accuracy of IOL power with mean absolute error (MAE) of 0.23 D^13^.

ANTERION has also been reported to show good repeatability in ocular biometry. Schiano-Lomoriello et al.^14^ reported COV < 1% for corneal diameter, while for CCT, ACD, LT and AL measurement, ICC was 0.98. Another study group investigated the repeatability of CCT, ACD, LT, and AL and revealed ICC > 0.92^15^ and WTW, angle opening distance, spur to spur, and trabecular iris space area with ICC > 0.97^16^. In addition, they reported repeatability of the whole corneal parameters with ICC > 0.98^17^. Pardeshi et al.^18^ reported the repeatability of the anterior chamber parameters with ICC of 0.97–0.99. In view of this good performance of the two devices, they can both be employed for IOL calculation.

In recent years, the former SS-OCT biometer, IOLMaster700 (Carl Zeiss Meditec, CA), has been widely used for IOL calculation. Interestingly, the newer SS-OCT, ANTERION, has developed some new modules for ocular biometry including cornea, cataract, metrics and imaging applications. With these advantages, ANTERION could possibly become the new standard ocular biometer.

In the present study, ANTERION demonstrated excellent correlation with G6 in all parameters, with ICC > 0.8. Almost all the parameters were arithmetically different, but the numbers were small and seemed to be non-significant for the purposes of clinical practice. For instance, AL, a key factor for IOL calculation, was 0.03 mm different with ICC 0.999. With this mean AL difference, IOL power difference was approximately 0.22 D which could be considered negligible for the purposes of IOL power selection^19^. Even though the devices use different OCT platforms to assess AL, they yield good correlation in IOL calculation. Theoretically, SS-OCT appears to be superior to OLCI in terms of finer resolution, higher scan rate and shorter acquisition time; however, this real-world data demonstrated that they had comparable outcomes in IOL calculation.

The IOL powers calculated by the 2 devices were within 1 D in 90 from 96 eyes (93.75%), with prediction error between the devices of within 0.03 D. These results show that both devices can provide an acceptable range of IOL power; however, the various parameters were not interchangeable.

In another comparative study of IOL calculation between SS-OCT and GSA: IOLMaster 700 and G6, Jung et al. reported good repeatability and comparable mean ACD, LT and AL. AL was 0.01 mm different between the devices and the prediction error was within 1 D in 100% of cases; however, significant differences were found in mean keratometry^2^. Their evaluation of 40 postoperative PEI eyes revealed a mean absolute error (MAE) within 1 D in 100% of cases. Further evaluation of MAE after PEI in our study is needed to validate the performance of ANTERION in clinical practice.

Regarding biometric measurement failure, previous studies have reported the rate from IOLMaster 700 of 0–7.4%^4,20^ and from G6 of 6.5%^2^. In our study, the IOL calculation could not be obtained with G6 in 2 (1.9%) eyes and with ANTERION in 4 (3.9%) eyes, which is in range of the findings of previous studies. All those 6 eyes failed to obtain AL and were excluded from analysis. Interestingly, the excluded patients did not have dense cataract, unlike the cases reported by previous studies^20^, but rather the failures were related to lack of cooperation during scanning. Ocular surface disease (OSD) in these patients could have triggered the occurrence of frequent blinking, resulting in failure to obtain AL. Clinicians should be aware of this condition; furthermore, proper management of OSD before scanning would improve the image quality and accuracy of the biometry^20–22^.

### Limitations

The limitations of the present study included the fact that we used only Barrett formula for IOL calculation and one IOL model of SN60WF, which might not be representative of the other ones. We did not exclude glaucoma patients, who could have had ocular surface disease from topical anti-glaucoma drops, and this may have affected IOL biometry. The Lens Opacity Classification system was not evaluated for those patients; hence, we cannot be sure to what extent the devices penetrated the various type of cataracts. Finally, the present study did not have results for MAE post PEI, and further evaluation of this issue is an ongoing project in our institute.

## Conclusions

To the best of our knowledge, this is the first comparison of the performance of ANTERION and G6 in ocular biometry and IOL calculation. Bland–Altman plots demonstrated excellent agreement between the devices, and comparison of biometry revealed an acceptable calculated IOL power within 1 D in 92.7% of cases. Biometries of the devices were arithmetically different; however, the mean differences of the key factors in IOL calculation were small and appeared to be negligible for the purposes of clinical application. Although the two devices achieved comparable biometric measurements, they cannot be used interchangeably.

## Supplementary Information

Supplementary Information.

## Data Availability

The data supporting the findings in this study are available in the main text, table, and figures. Raw dataset can be accessed in supplementary data.
