# Supplementary material for: Clinical evaluation of ocular biometry of dual Scheimpflug analyzer, GALILEI G6 and swept source optical coherence tomography, ANTERION
Source: Sci Rep. 2022 Mar 4;12:3602. doi: 10.1038/s41598-022-07696-1 (PMC8897498; doi:10.1038/s41598-022-07696-1)
Supplement: Supplementary file 1 — Supplementary Information. [file 41598_2022_7696_MOESM1_ESM.pdf]

Clinical Evaluation of Ocular Biometry of Dual Scheimpflug Analyzer, GALILEI G6 and Swept Source Optical Coherence Tomography, ANTERION

Boonsong Wanichwecharungruang, MD[1,2]

Anyarak Amornpetchsathaporn, MD[1]

Kittipong Kongsomboon, MD, MBA, PhD[3]

Wisakorn Wongwijitsook, MD[1]

Kornkamol Annopawong, MD[1]

Somporn Chantra, MD[1*]

[1]Department of Ophthalmology, Rajavithi Hospital and Rangsit Medical College, Bangkok, Thailand.

[2]Department of Ophthalmology, Priest Hospital, Bangkok, Thailand.

[3]Department of Preventive and Social Medicine, Faculty of Medicine, Srinakharinwirot University, Nakhon Nayok, Thailand.

Running title: Evaluation of ocular biometry of ANTERION and GALILEI G6 .

*Corresponding author: Somporn Chantra, MD.

Department of Ophthalmology, Rajavithi Hospital,

2, Phayatai Road, Bangkok,

Thailand 10400.

Tel: 66 86-541-3765

Email: chantrasomporn@yahoo.com

Keywords: Ocular biometry, optical biometer, swept source OCT, Scheimpflug camera, agreement, IOL calculation, cataract, Bland-Altman plot

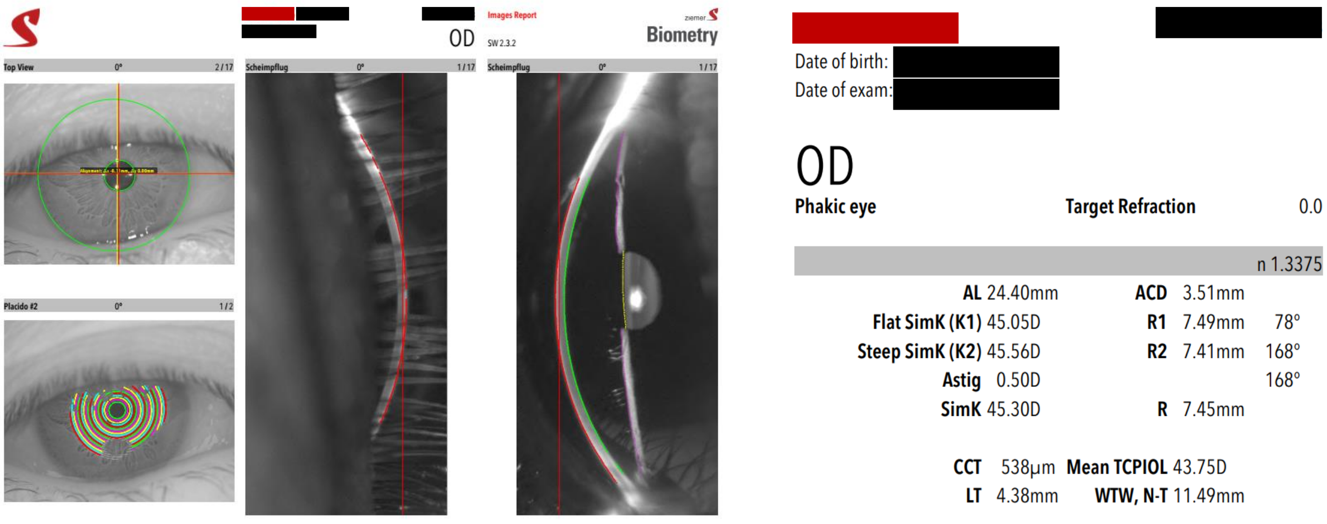

Supplementary figure 1: Ocular biometry reported from GALILEI G6 of the right eye of a 71 year-old Thai male with diagnosis of senile cataract.

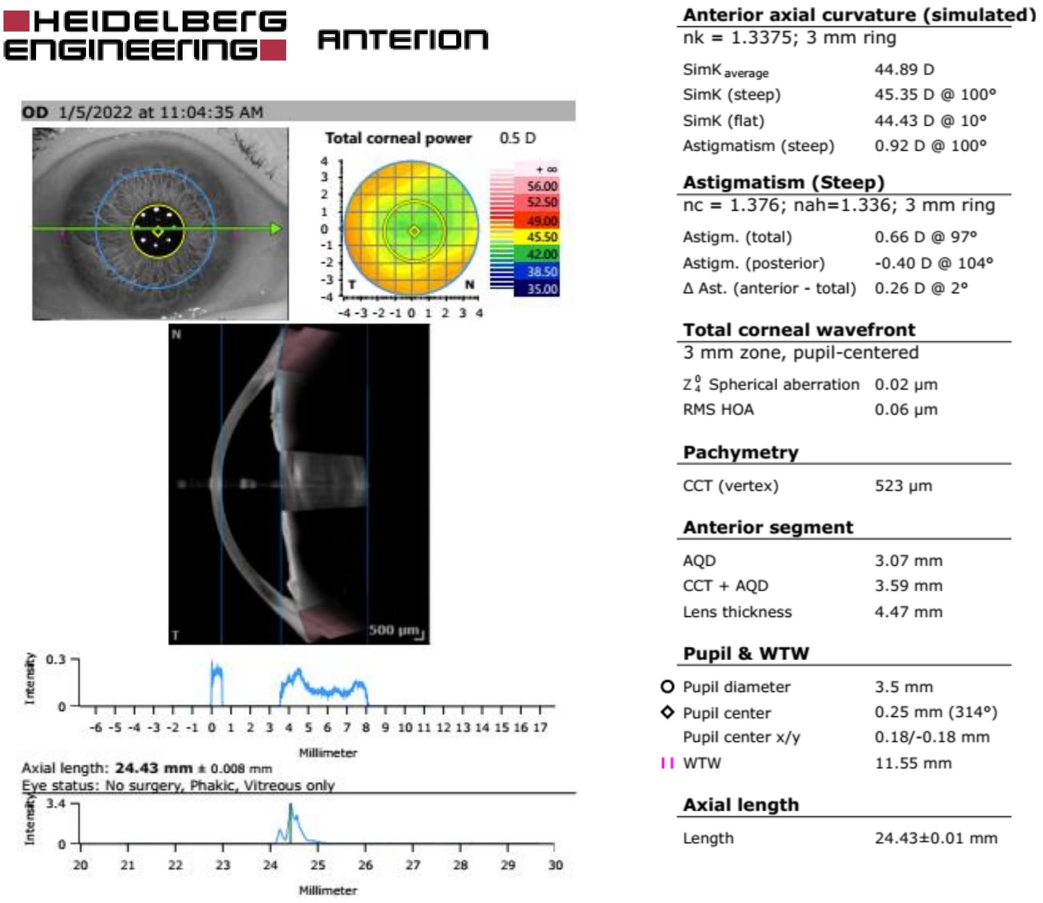

Supplementary figure 2: Ocular biometry reported from ANTERION of the same eye and same patient as the supplementary figure 1.

Supplementary figures were created by MICROSOFT POWERPOINT for MICROSOFT 365 MSO (Version 2112).
